# Séries temporelles: déterminants pathologiques des examens cytobiochimiques d’urines et infection urinaire entre 2011-2014 aux cliniques universitaires de Kinshasa

**DOI:** 10.11604/pamj.2021.40.211.29330

**Published:** 2021-12-08

**Authors:** Jacques Bikaula Ngwidiwo, Mireille Solange Nganga Nkanga, Vandersal Salaboni Munzengi, Eugène Epombo, Yvon Wangi Ngoy, Héritier Mawalala Malengele, Etienne Mokondjimobe, Benjamin Longo Mbenza

**Affiliations:** 1 Department of Medical Biology, Kinshasa University Clinics, Faculty of Medicine, Kinshasa, Democratic Republic of Congo,; 2 Lomo University of Research, Kinshasa, Democratic Republic of Congo,; 3 Marien Ngouabi University, Brazzaville, Republic of Congo,; 4 Walter Sisulu University, Mthatha, South Africa

**Keywords:** Examens cytobiochimiques, infection urinaire, sexe féminin, saisons de pluies, Cytobiochemical tests, urinary tract infection, female sex, rainy seasons

## Abstract

**Introduction:**

l´examen cytobiochimique urinaire est un outil complémentaire le plus demandé au laboratoire à côté de l´hémogramme. Il a une grande valeur prédictive dans les infections urinaires quand il est correctement fait et scrupuleusement interprété. L´objectif de cette étude était d´évaluer l´ampleur, l´évolution, les déterminants, et les comorbidités cytobiochimiques de l´infection urinaire.

**Méthodes:**

il s´est agi d´une étude documentaire, avec des approches, descriptive, analytique et comparative portant sur des patients référés pour examens cytobiochimiques des urines aux laboratoires de Cliniques Universitaires de Kinshasa (CUK) entre 2011 et 2014.

**Résultats:**

au total, 8926 analyses cytobiochimiques ont été demandées avec moins de 2% d´analyses biochimiques. Les femmes étaient plus représentées (6426 femmes vs 2500 hommes) avec un sex ratio 3F: 1H. Il y avait plus de demandes dans la tranche d´âge de 30-39 ans (17%; n=1517). Les analyses ont plus été demandées pendant les saisons de pluies 72% (n=3511) avec le pic pendant les mois de mai. Les infections urinaires estimées à 54,8% [n=4892 ajusté dont E. coli (n=1937), Klebsiella (n= 993)] étaient plus diagnostiqués pendant la période de 2012 à 2014. Il existait une association indépendante et significative entre le sexe féminin (OR aj = 3,5; IC = 95%; 3,1-3,8; P<0,0001, l´admission pendant les saisons de pluies (OR aj = 1,3, IC = 95%; 1,2-1,4; P<0,0001 et l´infection urinaire.

**Conclusion:**

l´infection urinaire émerge comme une préoccupation majeure pour les patients de sexe féminin et admises pendant les saisons de pluies et les années 2012-2014 de la série de 2011 à 2014 aux CUK. La rareté de l´infection urinaire était observée au cours de l´année La Nina 2011 post-l´année trop chaude EL Nino alors que l´ampleur de l´infection urinaire était coexistante pendant les années 2012-2014 normales relativement chaudes pré-l´année très chaude El Nino 2015. Il existe donc une interaction entre le climat tropical chaud et humide de la ville de Kinshasa et le climat global (mondial) froid dans le cadre de la variabilité climatique/réchauffement climatique, pouvant expliquer la flambée des infections urinaire à Kinshasa.

## Abstract (en)

**Introduction:**

cytobiochemical analysis of urine samples is the most requested complementary laboratory tool along with blood count. It has high predictive value in patients with urinary tract infections when it is properly done and scrupulously interpreted. The purpose of this study was to assess the rate, progression, determinants and cytobiochemical comorbidities of urinary tract infections.

**Methods:**

we conducted a documentary, descriptive, analytical and comparative study of patients referred for cytobiochemical examinations of urine to the laboratories of the University Clinics in Kinshasa (UCK) between 2011 and 2014.

**Results:**

a total of 8926 cytobiochemical tests were requested, with less than 2% of biochemical tests. The study involved more women than men (6426 women vs 2500 men), with a sex ratio of 3F: 1M. Cytobiochemical tests were more requested in the 30-39-year age group (17%; n=1517) mand during the rainy seasons 72% (n=3511), with a peak in May. Urinary tract infections accounted for 54,8% [adjusted estimates n=4892 including E. coli (n=1937), Klebsiella (n=993)] and were mainly diagnosed over the period 2012-2014. There was an independent and significant association between female sex (adjusted OR = 3.5; CI = 95%; 3.1-3.8; P<0.0001), admission during the rainy seasons (adjusted OR = 1.3; CI = 95%; 1.2-1.4; P<0.0001) and urinary tract infection.

**Conclusion:**

urinary tract infection was a major concern for female patients admitted during the rainy seasons and over the years 2012-2014 at the UCK. Urinary tract infections were rare over the Nina year 2011 after the hottest El Nino year, while the rate of urinary tract infections was the same over the years 2012-2014, which were relatively hot before the the hottest El Nino year 2015. This study highlights that there is an interaction between the hot and humid tropical climatic conditions of the city of Kinshasa and the global cold climate, in the context of climate variability, global warming, which may explain the outbreak of urinary infections in Kinshasa.

## Introduction

L´examen cytobiochimique urinaire est un outil complémentaire le plus demandé au laboratoire à côté de l´hémogramme en Europe. Cet examen a une grande valeur prédictive (orientation) dans les infections urinaires quand il est correctement fait et scrupuleusement interprété [1].

La présence d´autres marqueurs biologiques dans les urines (cytobiochimie: glucose, cristaux, cylindres, bilirubine, hématurie) oriente plus vers d´autres entités cliniques non transmissibles dont la goutte, le diabète sucré, les maladies rénales chroniques et les cancers [1]. En effet, le diagnostic de ces entités cliniques peut être orienté par l´examen urinaire. Cependant cet examen est sujet à multiples facteurs, anatomiques, biologiques, métaboliques et physicochimiques, environnementaux et demande une technicité et une interprétation correcte des résultats. Bien conduit, l´examen cytobiochimique apporte la présence des cylindres épithéliaux et granulaires, des cylindres hyalins, les érythrocytes, les leucocytes, la protéinurie, l´hématurie qui peuvent déjà donner une valeur négative et orienté le clinicien [2-4]. C´est ainsi qu´une hématurie est significative dans le cancer de la vessie, les cellules épithéliales dans les pathologies tubulaires, les cylindres hématiques dans les glomérulopathies, la bactériurie, la leucocyturie dans les cystites et les pyélonéphrites et les levures dans les infertilités secondaires [5].

Au regard de ces éléments, l´examen cytobiochimique des urines a une grande valeur prédictive dans l´orientation du diagnostic de la comorbidité de l´infection urinaire et d´autres pathologies non transmissibles [4,6]. Comme rapporté dans le monde en général [7] et dans certains pays d´Afrique subsaharienne en particulier [8,9], la prise en charge des examens d´urines est essentiellement focalisée sur les infections, il en de même aux Cliniques Universitaires de Kinshasa (CUK), République Démocratique du Congo (RDC) région centrale de l´Afrique subsaharienne. Par impression bioclinique aux CUK, il est noté une augmentation de demande d´analyses cytobiochimiques d´urines avec le temps (séries temporelles ou séries chronologiques) et au regard de l´émergence des maladies non transmissibles dont le diabète sucré, les maladies rénales chroniques, le cancer, et les maladies oculaires [10,11].

En outre, la prise en charge scientifique (étiologie, diagnostic clinique, diagnostic de laboratoire , traitement, pronostic, et prévention) de l´infection urinaire aux CUK, est complexe pour les raisons suivantes: avancement en âge relatif des patients avec les maladies non transmissibles en Médecine interne [12,13], absence de collaboration (sous-utilisation de laboratoire de biologie clinique, existence des laboratoires intra départemental, référence extérieur des examens) entre les cliniciens et les biologistes hospitaliers, déficit d´un plateau techniques adéquat et inexistence des approches, des stratégies des standardisées des examens cytobiochimiques d´urines à cela greffé le manque de motivation et de formation continue du personnel de laboratoire. L´objectif de cette étude était d´évaluer l´ampleur, l´évolution, les déterminants, et les comorbidités cytobiochimiques de l´infection urinaire à travers une approche transversale documentaire sur des patients référés pour examens cytobiochimiques des urines aux laboratoires de CUK entre 2011 et 2014.

## Méthodes

**Nature et cadre de l´étude**: la présente étude est une revue documentaire, avec des approches descriptive, analytique et comparative portant sur des patients référés pour examens cytobiochimiques des urines aux laboratoires de CUK de 2011 à 2014. La présente étude a adopté les approches suivantes: étude documentaire, une analyse rétrospective (réalisée en 2018), une approche descriptive et approche comparative (association, corrélation). Elle a porté sur quatre années successives soit de 2011 à 2014. Le laboratoire de Biologie clinique des Cliniques Universitaires de Kinshasa a servi de cadre à cette étude.

**Conception de l´étude**: les modèles conceptuels se fondaient sur un cadre théorique (connaissances générées) partant des facteurs exogènes et endogènes (variables proximales indépendantes déterminants pour expliquer la fréquence des examens cytobiochimiques d´urines et des infections urinaires selon la tendance temporelle. Ainsi la question de recherche était la suivante: est-il une croissance des troubles cytobiochimiques et infectieux urinaires de 2011 à 2014? C´est pourquoi les modèles mathématiques a testé plusieurs hypothèses suivantes; a) il existe une association significative entre le sexe féminin et les saisons; b) il existe une association significative entre.

Cette étude a concerné les résultats des analyses cytobiochimiques des patients référés aux laboratoires de Biologie Clinique des CUK. Il s´est agi d´un échantillonnage exhaustif des registres des résultats d´analyses cytobiochimiques d´urines de la période d´étude. Seuls les registres qui avaient les résultats de tous les mois de l´année ont inclus dans la présente étude. Les paramètres d´intérêts étaient les variables démographiques/facteurs non modifiables (sexe et âge) les années et mois d´admission (chrono-biologie) les résultats microscopiques (CE, globules blancs (GB), globules rouges (GR), cylindres cristaux), les résultats biochimiques (glycosurie qualitative et protéinurie qualitative/albuminurie). L´analyse microscopique et les méthodes de Fehling et à ébullition ont été utilisés respectivement pour le sédiment urinaire, la recherche qualitative de glucose et la recherche qualitative des protéines. L´interprétation des résultats des sédiments urinaires a été faite selon la technique de Schumann. Pour les analyses biochimiques l´interprétation du résultat s´est fait selon le changement de coloration du mélange urine-réactif. Chaque coloration correspond à une échelle de valeur.

**Analyses statistiques**: des analyses univariées et multivariées ont été effectuées dans la présente étude. En analyse uni variée, les données catégorielles (qualitatives = nominales) ont été présentées sous-forme de nombre (n) ou fréquence et sous-forme de proportions (%). Les données quantitatives étaient représentées sous-forme de moyennes, écart-types ou médiane (minimum, maximum) selon quelles étaient normalement distribuées ou non. En analyse multivariée, la régression logistique binaire (analyse des réponses de type dichotomique: présence vs absence) a été utilisée pour des variables dépendantes expliquées par des variables indépendantes selon la stratégie pas à pas antérograde (Stepwise Method forward) et après ajustement pour certains facteurs de confusion.

**Considération éthique**: le protocole de ce mémoire a reçu l´approbation du Comité Ethique sous le numéro 110/CNES/BN/PMMF/2019.

## Résultats

**Figure 1:**
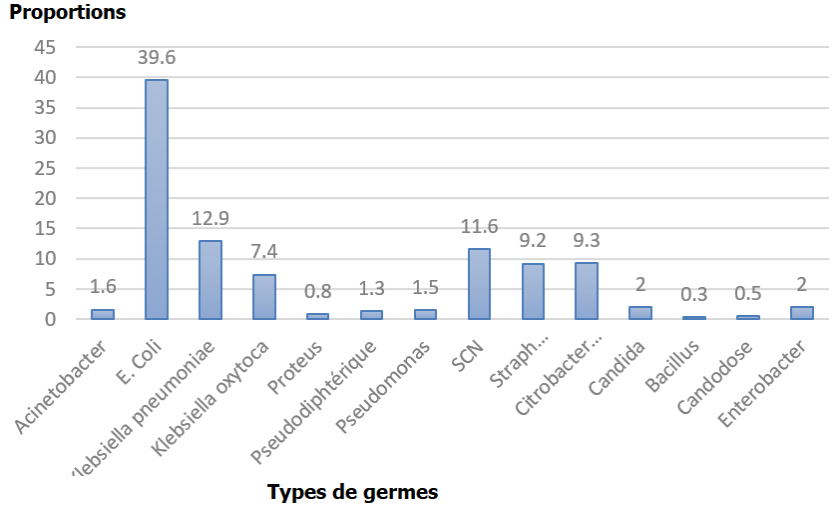
répartition des proportions des analyses microbiologiques selon les types de germes dont *E. Coli* avec allure épidémique

**Figure 2:**
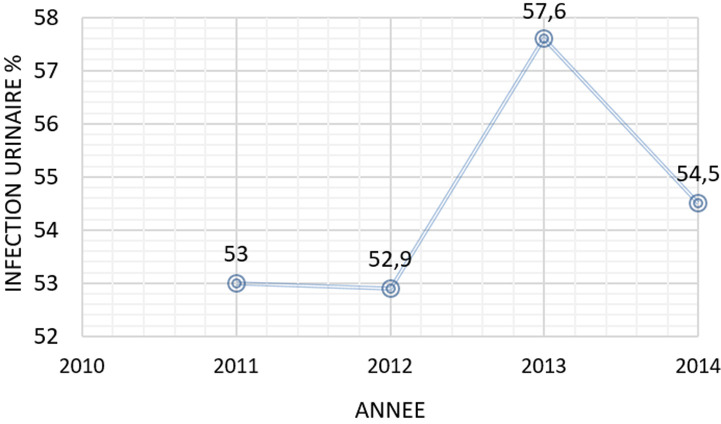
répartition de l´infection urinaire selon les années/variabilité climatique en analyse univariée

**Figure 3:**
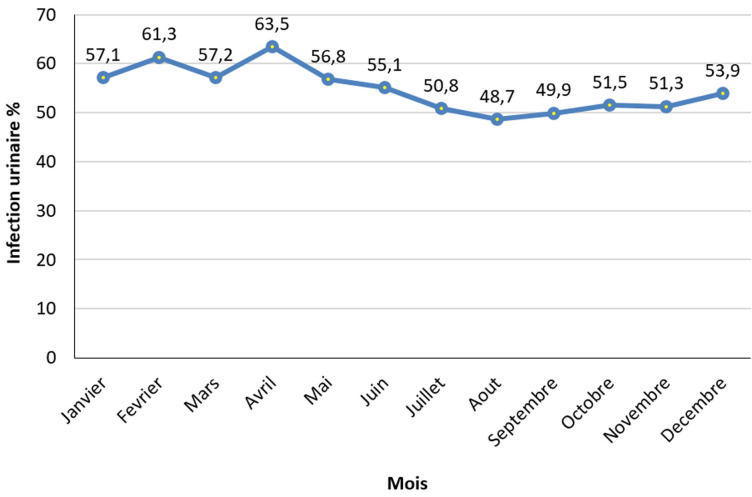
répartition de l´infection urinaire selon les mois/climat local en analyse univariée

Au total 8926 examens cytobiochimiques d´urine ont été successivement analysés de 2011-2014. Il y avait une surreprésentation du sexe féminin (n= 6426 femmes vs n = 2500 hommes): avec un sex ratio de 3F: 1H. La tranche d´âge de 30 à 39 ans (n=1517) était la plus représentée. Cette étude a montré plus d´examens cytobiochimiques d´urines pathologiques (n=4910) que d´examens cytobiochimiques d´urines normaux (n = 4016). Le département de médecine interne (n=3750) et le département de gynéco-obstétrique (n = 2276) ont le plus référé les patients pour les analyse cytobiochimiques. Le pic des analyses microbiologiques était observé pour *E. Coli* (Figure 1). Il y avait moins de demande de recherche d´albuminurie et de glycosurie. La microscopie urinaire pathologique était plus significative que la microscopie normale au cours de la présente étude. La présente étude a montré une forte et significative (P<0,05) représentation d´infection urinaire pendant l´année 2013 et un faible taux pendant les années 2011 et 2012 (Figure 2). Avec des pics de d´infection urinaire pendant les mois d´avril et des faibles taux pendant les mois d´août, la différence statistique étant significative (Figure 3).

Parmi les sédiments urinaires pathologiques (n=4892 ajustés), il a été observé la prédominance *d´E. coli* (n=1937) et de *Klebsiella pneumoniae* (n=631), contre la rareté par ordre décroissant de SCN (n=567), de *Citrobacter* (n=455), de Klebsiella oxytoca (n=362), de *Staphylococcus épidermidis* (n=256), de *Pseudo-diphtéria* (n=136), *Acinectobacter* (n=72), *Staphylococcus areus* (n= 56), *Proteus mirabilis* et *vulgaris* (n=40) et *Bacillus* (n=16) à l´ examen cytobactériologique des urines (ECBU) (Figure 1). Excepté le taux de cristaux d´oxalate de calcium qui avoisinait les 10% en microscopie optique du sédiment urinaire, les autres troubles biochimiques observées en microscopie optique (présence des cylindres, cristaux d´oxalate, cristaux de sulfate, cristaux de phosphate d´ammoniaque et cristaux d´urates) et les troubles biochimiques détectés respectivement par la méthode d´ébullition de protéine et de Fehling pour le glucose étaient rares en présence de l´infection urinaire.

Il existait une association uni variée significative entre le sexe féminin et les infections urinaires, la tranche d´âge de 30-39 ans, le Département de Gynéco-Obstétrique et la saison de pluies. Au plan biochimique, l´albuminurie était observée chez 0,7 % des patients (n=61/8865) contre la glycosurie chez 1,3 % des patients (n=118/8808). En analyse multivariée, après ajustement pour les variables de confusion tel que l´âge en utilisant la régression logistique binaire, seuls le sexe féminin et l´admission pendant la saison de pluie et la croissance de l´examen cytobiochimique ont été les déterminants les plus importants, indépendants et significatifs de la présence (prévalence hospitalière) de la série temporelle de l´examen cytobiochimique et de l´infection urinaire aux CUK de 2011 à 2014 (Tableau 1).

**Tableau 1:** déterminants de l´infection urinaire en analyse multivariée

Variables	B	S.E.	Wald	ddl	P	Exp(B) (OR aj)	CI 95%	
							Lower	Upper
**Genre**	1,241	0,050	622,059	1	<0,0001	3,460	3,138	3,814
**Saisons**	0,239	0,048	24,834	1	<0,0001	1,270	1,156	1,396

## Discussion

Il s´agit de la première étude aux CUK/DRC a analysé scientifiquement les relations entre les variables indépendantes ou explicatives dont les séries temporelles (mois, années, saisons/climat local), certains facteurs associés/déterminants (sexe, âge, départements médico-chirurgicaux), et les variables dépendantes ou expliquées (l´infection par sédiment urinaire/leucocyturie, cellule épithéliale, bactéries, levures, l´albuminurie et la glycosurie qualitatives, la cylindriurie, les cristaux d´oxalate de calcium, cristaux de phosphate de calcium, cristaux de sulfate, les cristaux de phosphate d´ammoniaque, cristaux d´urates et l´hématurie). La discussion a insisté sur les variations du nombre des examens cytobiochimiques des urines, de l´infection urinaire, et des troubles cytobiochimiques en fonction du temps.

**Influence des séries temporelles**: la présente étude a montré une variation significative des proportions des examens cytobiochimiques des urines selon les années d´admissions, les mois et les saisons. En effet, deux périodes des années d´admissions des patients référés pour ces examens se sont successivement démarqués par la rareté d´examens au cours de l´année 2011 considérée année La Nina (extrémité de la saison froide de la variabilité climatique dans le cadre du changement climatique /réchauffement planétaire) en comparaison à des proportions dix fois plus élevées entre 2012 et 2014 années consécutives normales dans le cadre du changement climatique et relativement chaudes[10,13,14]. Ces résultats étaient obtenus entre 2010 et 2015 années El Nino très chaudes dans le cadre de la variabilité climatique et du réchauffement planétaire. En ce qui concerne le climat local de la ville de Kinshasa, RDC, cadre de cette étude, il a plutôt été observé un excès de réalisation des examens cytobiochimiques urinaires et cela, pendant la grande saison de pluies (mois d´avril et de mai pendant lequel on a noté un pic très chaud). Par contre une sous réalisation a été observée au cours de la grande saison sèche froide entre les mois de juin et septembre (nadir) [14].

Dans une approche dichotomique sinon binaire pour présence contre absence de l´infection urinaire dans la présente étude, comparées aux saisons (petite +grande) sèches, les saisons (petite et grande) de pluies étaient un temps plus délétères en faveur de l´infection urinaire, confirmant ainsi les résultats de la littérature [10,12,13,15-17]. La littérature rapporte que l´incidence des infections urinaires augmente en été qu´en hiver [15]. En plus, une étude réalisée en France a relevé l´augmentation de la vente des médicaments contre les infections urinaires non seulement en France, mais également aux USA, en Chine ainsi qu´en Italie [17]. Ces deux saisons sont superposables aux saisons sèches et saisons de pluies telles que définies en Afrique sub-saharienne en général et en RDC en particulier. En effet, les saisons de pluies à Kinshasa sont caractérisées par de fortes précipitations, de l´humidité et de fortes températures. Les interactions entre ces trois paramètres peuvent aisément expliquer la forte proportion de cas d´infection urinaire observés dans la présente étude [16,17].

### Variables sociodémographiques et examens cytobiochimiques d´urines

**Sexe**: comme il fallait s´y attendre, la prédominance féminine était caractéristique de l´ensemble des examens cytobiochimiques et de la présence de l´infection urinaire dans la présente étude. En effet, le sexe féminin a conféré un triple risque univarié de l´infection urinaire dans cette étude. Plusieurs raisons dont l´anatomie (la courte longueur de l´urètre, la courte distance entre l´anus et le méat urétral, la permissivité des environnements vaginal et périnéal à la colonisation microbienne), la physiologie de l´appareil urogénital féminin, l´hygiène, la promiscuité, la grossesse, le Département de Gynéco-Obstétrique et la ménopause expliquent la susceptibilité (prédisposition) du sexe féminin à l´infection urinaire. En outre, comme décrit dans d´autres études, la carence hormonale perturbe la flore vaginale, favorise la disparition des lactobacilles et alcalinise le pH favorisant ainsi, la susceptibilité à l´infection urinaire par des souches uropathogènes chez les femmes (sujets âgés) [18-22].

**Âge**: contrairement aux données de la littérature qui démontrent que l´infection urinaire est associée à l´avancement en âge, dans la présente étude, l´infection urinaire était (vieillissement) [19,20] associée à l´âge jeune et actif. En effet, les maladies non transmissibles (MNT) notamment, le diabète sucré, les maladies rénales chroniques qui sont l´apanage des personnes âgées se compliquent souvent par les infections urinaires [20,22].

**Coexistence des troubles biochimique de l´urine jet de l´infection urinaire**: dans la présente étude, l´examen microscopique du sédiment urinaire chez des patients avec infection urinaire était caractéristique de la présence des différents types de cristaux suggestifs de lithiase urinaire [23-25], de l´albuminurie suggestive des maladies rénales chroniques [11,13], de la glycosurie suggestive du diabète sucré [11,26] et de l´hématurie suggestive d´un cancer de la vessie, de la prostate ou d´une lésion de l´arbre urinaire [1]. En effet, l´infection urinaire est associée à la diminution de l´immunité, surtout de l´immunité innée, facteur prépondérant dans de la défense de l´hôte [27-29], et qu´en cas d´hématurie et de la présence des cristaux d´oxalates de calcium on assiste à une stase urinaire et une pullulation microbienne [26]. La coexistence des troubles biochimiques de l´urine et de l´infection urinaire souligne la transition nutritionnelle liée à une prise excessive des aliments trop riches en protéines, en calorie et en sel [26] caractéristique du diabète sucré. Par ailleurs, l´excès de diète riche en produit laitier, en poissons et en certains légumes (aubergine) favorise la présence des cristaux d´oxalate de calcium.

**Déterminants indépendants de l´infection urinaire**: parmi les trois facteurs associés à l´infection urinaire, tels que la tranche d´âge de 30-39 ans, le sexe féminin et l´admission durant la saison des pluies, seuls le sexe féminin et l´admission pendant les saisons de pluies étaient maintenus par le modèle multivarié en régression logistique comme déterminants indépendants importants et significatifs du risque élevé d´infection urinaire dans la présente étude. Ces derniers résultats de la population Kinoise (RDC) corroborent les autres évidences de la littérature [15,16,18-22]. L´âge jeune était plutôt une variable de confusion dans la présente population en phase pré-transition démographique en activité sexuelle incriminée dans les infections sexuellement transmissibles, et dans la diminution de la défense (IgA) et l´infection urinaire.

**Implications de l´étude**: les résultats de la présente étude seront utilisés pour la recherche et la formation continue. Il est donc important de se focaliser sur le renforcement de capacité du personnel de Biologie Clinique et sur les implications à caractère préventif de l´infection urinaire en général et chez les femmes admises au cours des saisons des pluies.

**Forces et limites de l´étude**: la présente étude a présenté des forces balancées par certaines limites à un certain degré. Les mérites de la présente étude ont été soulignés par une très grande taille de la population examinée avec une rigueur scientifique à travers des résultats non encore produits en République Démocratique du Congo (RDC). Par contre, les faiblesses de cette étude sont inhérentes à sa nature secondaire et documentaire souvent caractérisée par des biais de complétude des informations tels que l´absence des caractéristiques physico-chimiques de l´urine dans l´ensemble des bulletins d´analyses aux cliniques universitaires de Kinshasa; caractéristiques souvent obtenues par la simple utilisation des bandelettes urinaires multiparamétriques. Ainsi, l´audit clinique veillera à ce que le Service de Biologie Clinique des CUK mentionne le volume, la couleur, la limpidité, l´odeur, la densité, le pH et associe aux méthodes déjà utilisées, les bandelettes urinaires multiparamétriques mais aussi d´introduire d´autres méthodes automatisés de cytobiochimie urinaire.

## Conclusion

La présente étude a démontré que l´infection urinaire constitue une préoccupation importante de niveau problème de santé publique aux Cliniques Universitaires de Kinshasa. En plus, elle a émergé comme une préoccupation majeure pour les patients de sexe féminin admis pendant les saisons de pluies et les années 2012- 2014 de la série de 2011 à 2014 aux CUK. La rareté de l´infection urinaire était observée au cours de l´année La Nina 2011 post l´année trop chaude El Nino 2010 très chaude (extrémité de la saison froide de la variabilité climatique dans le cadre du changement climatique/réchauffement planétaire) alors que l´ampleur de l´infection urinaire était coexistante pendant les années 2012-2014 normale relativement chaudes pré- l´année très chaude El Nino 2015 dans le cadre du changement climatique/réchauffement planétaire. Il existe donc une interaction entre le climat tropical chaud et humide de la ville de Kinshasa et le climat global (mondial) froid dans le cadre du phénomène El Nino-La Nina/variabilité climatique/réchauffement climatique, pouvant expliquer la flambée des infections urinaires à Kinshasa. Conflit d´intérêt: les auteurs n´ont signalé aucun conflit d´intérêt

### Etat des connaissances sur le sujet

- L´examen cytobiochimique des urines a une grande valeur prédictive dans l´orientation du diagnostic de la comorbidité de l´infection urinaire et d´autres pathologies non transmissibles;
- Cet examen est sujet à multiples facteurs, anatomiques, biologiques, métaboliques et physicochimiques, environnementaux et demande une technicité et une interprétation correcte des résultats;
- La présence d´autres marqueurs biologiques dans les urines (cytobiochimie: glucose, cristaux, cylindres, bilirubine, hématurie) oriente plus vers d´autres entités cliniques non transmissibles dont la goutte, le diabète sucré, les maladies rénales chroniques et les cancers.

### Contribution de notre étude à la connaissance

- La première étude aux CUK/RD Congo a analysé scientifiquement les relations entre les variables indépendantes ou explicatives dont les séries temporelles (mois, années, saisons/climat local), certains facteurs associés/déterminants (sexe, âge, départements médico-chirurgicaux), et les variables dépendantes;
- Seul le sexe féminin et l´admission pendant les saisons de pluies étaient maintenus par le modèle multivarié en régression logistique comme déterminants indépendants importants et significatifs du risque élevé d´infection urinaire dans la présente étude;
- Les mérites de la présente étude ont été soulignés par une très grande taille de la population examinée avec une rigueur scientifique à travers des résultats non encore produits en République Démocratique du Congo (RDC).
